# Preclinical detection of infectivity and disease-specific PrP in blood throughout the incubation period of prion disease

**DOI:** 10.1038/srep17742

**Published:** 2015-12-03

**Authors:** Elizabeth B. Sawyer, Julie Ann Edgeworth, Claire Thomas, John Collinge, Graham S. Jackson

**Affiliations:** 1 MRC Prion Unit, Department of Neurodegenerative Disease, UCL Institute of Neurology, Queen Square, London WC1N 3BG, UK

## Abstract

Variant Creutzfeldt-Jakob disease (vCJD) is a fatal neurodegenerative disorder characterised by accumulation of pathological isoforms of the prion protein, PrP. Although cases of clinical vCJD are rare, there is evidence there may be tens of thousands of infectious carriers in the United Kingdom alone. This raises concern about the potential for perpetuation of infection via medical procedures, in particular transfusion of contaminated blood products. Accurate biochemical detection of prion infection is crucial to mitigate risk and we have previously reported a blood assay for vCJD. This assay is sensitive for abnormal PrP conformers at the earliest stages of preclinical prion disease in mice and precedes the maximum infectious titre in blood. Not only does this support the possibility of screening asymptomatic individuals, it will also facilitate the elucidation of the complex relationship that exists between the ensemble of abnormal PrP conformers present in blood and the relationship to infectivity.

Creutzfeldt-Jakob disease (CJD) is an incurable neurodegenerative disorder characterised by the accumulation of pathological isoforms of the prion protein, PrP^1^,^2^. CJD may arise sporadically or be acquired through exposure to prions via contaminated surgical instruments or infected tissue, including dietary exposure to bovine spongiform encephalopathy (BSE) agents resulting in variant CJD (vCJD)^3^. As a consequence of widespread exposure to BSE prions via the UK food chain, it is thought that as many as 1 in 2000 of the population may be carriers of abnormal PrP isoforms^4^. Significant questions remain over the link between observation of these protein deposits in lymphatic tissue and the likelihood of developing vCJD, given that the relationship between infection in the lymphoid system and the brain is not clear and that the incubation period of the disease can be decades^5^,^6^.

The possibility that there are silent carriers of vCJD within the population is a cause for concern not only for the individuals affected but also because of the potential for perpetuation of vCJD infection via medical and dental treatments, particularly the transfusion of contaminated blood products. Several animal studies have demonstrated that prion transmission can occur by blood transfusion^7^,^8^ and that this is an extremely efficient route of infection^9^. Experimental observations have been echoed by confirmed secondary vCJD infections in humans who received blood products from apparently healthy donors who later developed prion disease^10^,^11^,^12^. This strongly suggests the presence of vCJD infectivity in blood and therefore the need for precautionary measures to prevent further infections, ideally to include testing for prion infection as recommended by the recent UK House of Commons Science and Technology Select Committee enquiry into vCJD.

The introduction of a validated screening assay for sub-clinical vCJD carriers would offer significant protection but presents two major challenges. Firstly, the unavailability of samples from individuals known to be sub-clinical carriers of vCJD with which to validate any assay and secondly, the need to detect very low (in the femtomolar range) concentrations of prions that are likely to be found in the blood in the preclinical phases of disease^13^.

To address the first concern animal models of prion disease provide the only means by which preclinical samples can be generated. Models previously employed have typically used sheep experimentally infected with either scrapie^14^ or BSE^8^ to obtain appropriate samples which have confirmed that blood and blood components are infectious in asymptomatic stages of disease. The majority of analyses have utilised protein-misfolding by cyclic amplification (PMCA)^15^,^16^ to achieve qualitative detection of abnormal PrP. However, there have been notable examples of bioassay in sheep yielding the important conclusions that all components of blood are infectious^8^, in particular white blood cell fractions^14^, and that transfusion leads to a greater risk of infecting a host than direct inoculation into the central nervous system^9^.

Although the use of sheep models allows the handling and experimental transfusion of whole units of blood and fractionated products to assess the impact and relevance of blood processing protocols, such experiments are extremely time consuming with incubation periods for disease measured in years and with associated high costs. Importantly, for all large animal experiments, and indeed conventional rodent bioassay, quantitative information is difficult to obtain.

To address the question of whether the Direct Detection Assay (DDA) has a sensitivity appropriate for the detection of carrier states we chose to study wild-type CD-1 mice experimentally infected with the Rocky Mountain Laboratory (RML)^17^ strain of prions for which there are rapid, high precision quantitative cell-based assays for infectivity^18^. Whole blood was taken from these animals at various stages of disease incubation and compared with that taken from uninfected healthy controls using two assays, the DDA, which detects the presence of disease-specific, abnormal PrP conformers, and the Standard Steel Binding Assay (SSBA)^18^ which allows quantification of infectivity. The SSBA has been demonstrated to be 100-fold more sensitive than conventional rodent bioassay with the ability to detect RML-infected brain diluted 10^10^-fold, corresponding to a prion concentration less than or equivalent to 1 intracranial LD_50_ units/ml.

The DDA is a highly sensitive assay that utilises a solid-state binding matrix to capture and concentrate disease-associated prion proteins which are then detected immunologically^19^. The analytical sensitivity of DDA for vCJD is equivalent to a 10^10^–fold dilution of vCJD prion-infected brain when diluted in blood. This corresponds to a diagnostic sensitivity to patient samples of 71%, whilst being 100% specific for prion disease^20^. The lack of any samples from known sub-clinical patients has prevented us from determining if these assay characteristics are sufficient for preclinical detection. Here we describe the application of both DDA and SSBA to blood samples taken from wild-type CD-1 mice at defined time points throughout the incubation period for prion disease.

Analytical sensitivity with DDA was slightly lower than we have previously reported for human samples^19^ and yet was sufficient to detect infection at all time points of incubation tested. Not only were we able to demonstrate the ability to qualitatively detect the relatively low levels of abnormal PrP conformers associated with sub-clinical infection, but by use of the SSBA we were able to quantify the infectious titre in our samples. Combined use of DDA and SSBA allows us to begin to understand the complex relationship that exists between infectious titre and the wider ensemble of detectable abnormal PrP conformers associated with prion infection.

## Results

### Identification of prion infected rodent blood samples

We have previously described a blood-based assay capable of detecting abnormal isoforms of PrP associated with vCJD infection^19^ that has a nominal specificity of 100%^20^. The established performance characteristics of the assay make it potentially suitable for adaptation to screening applications such as testing donated blood units. One uncertainty has been the ability of the assay to detect preclinical infection with prion disease and the paucity of samples from known carriers of vCJD has made this impossible to determine directly. By using the RML strain of rodent prions in mice we can generate samples from defined time points throughout the incubation period for disease and verify the level of DDA-reactive material in blood is sufficiently abundant for detection prior to the onset of clinical disease.

**Figure 1:**
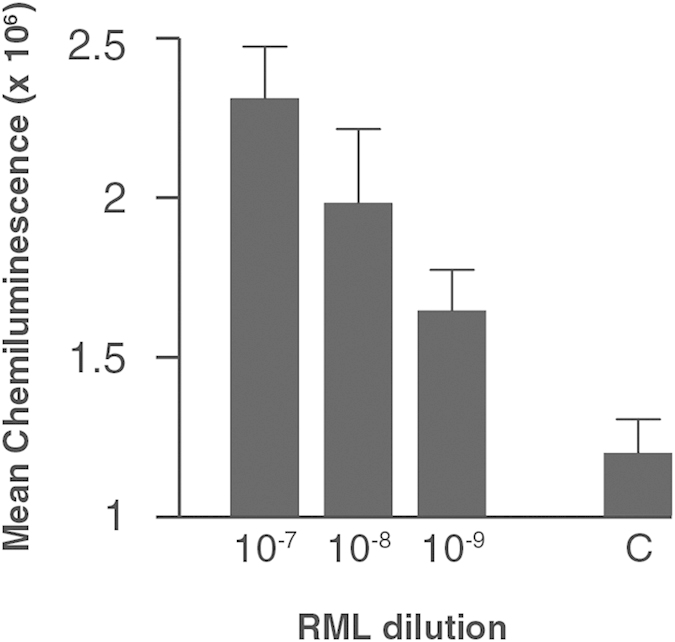
The detection limit for exogenous RML infection. A 10% w/v RML-prion infected brain homogenate was logarithmically diluted into whole blood collected from wild-type CD-1 mice down to 10^−9^ and analysed by DDA. Mean chemiluminescence signals with standard errors of the mean (SEM) are shown for two independent experiments with n = 3 for each experiment. The signal arising from the whole blood diluent alone is shown as a background control (C).

To establish assay sensitivity for RML prions we created exogenous spiked materials comprising RML prion-infected brain in whole blood from wild type (CD-1) mice. Infected samples could be clearly distinguished from control blood (Fig. 1) at a dilution of at least 10^9^-fold of brain (p = 0.02, unpaired t-test, two-tailed with Welch correction) a detection limit very similar to that we have previously determined for vCJD samples^19^. This limit of detection is sufficient for the diagnosis of patients and appropriate for use in public health risk assessment^20^.

**Figure 2:**
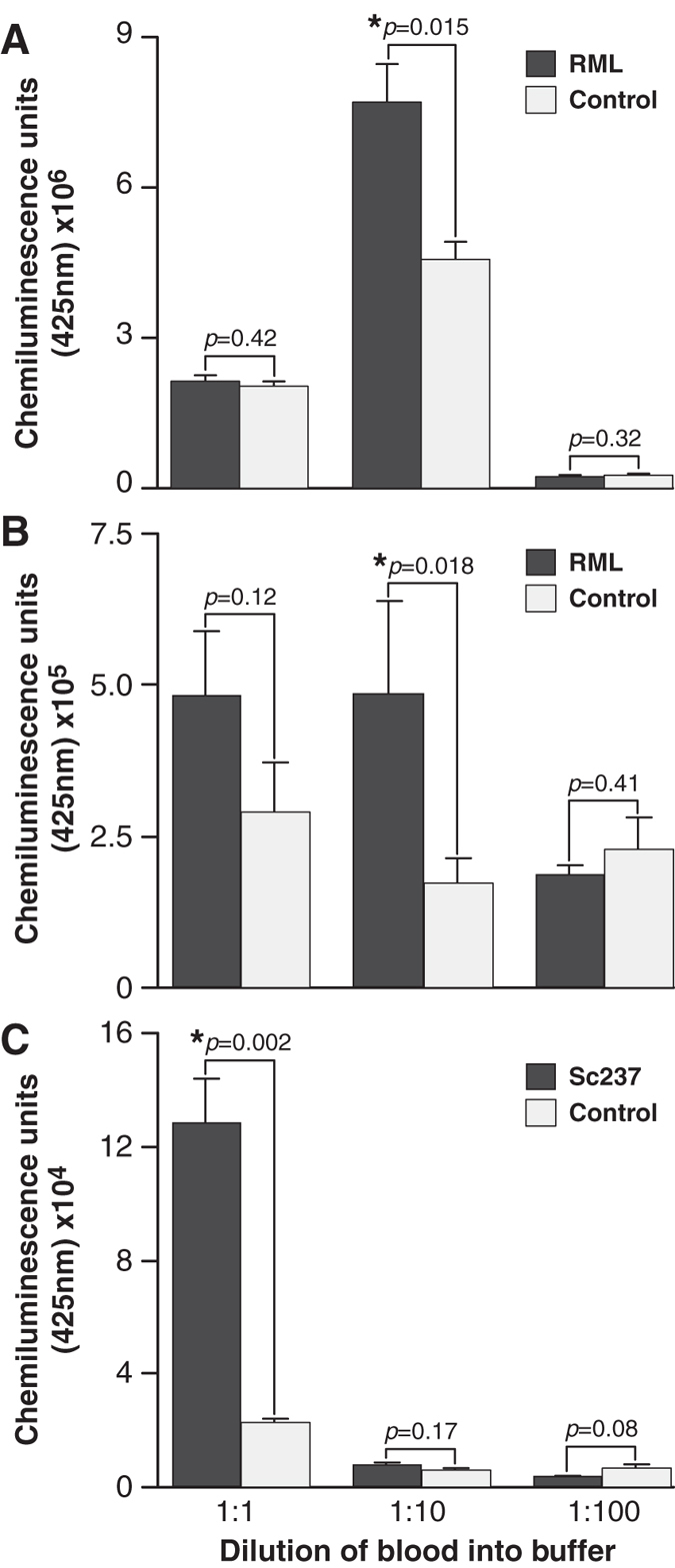
Discrimination of endogenous rodent infected blood from normal controls. Data shown are the mean chemiluminescence signals (arbitrary units) of pooled blood samples from prion-infected rodents at clinical endpoint (dark bars), compared to the mean signal from normal controls diluted (light bars). Blood samples were analysed at three dilution ratios to capture buffer; 1:1, 1:10 and 1:100. Panels (**A**,**B**) show signals from blood taken from CD-1 mice and Tga20 mice infected with RML prions compared to healthy controls, respectively. Panel (**C**) is data obtained from blood taken from Syrian hamsters infected with Sc237 prions compared to controls. Error bars represent the standard deviation from the mean signals (n = 3).

Examination of whole blood taken from wild-type CD-1 mice and Tga20 transgenic mice^21^, that overexpress murine PrP, infected with the RML prion strain at clinical end point as well as from hamsters infected with Sc237 prion strain was conducted by comparing to their relevant non-infected controls. The prion infected blood samples were examined at three different blood to buffer ratios, i.e. 1:1, 1:10 and 1:100 (Fig. 2). In each case the mean chemiluminescence signals from the prion-infected blood samples were significantly higher than those taken from healthy controls at an optimal blood to buffer ratio (p value < 0.05, unpaired t-test, two-tailed with Welch correction). The ratio of blood to buffer mix required to observe the maximum differential between infected and control samples was determined to be different for each of the prion strains examined and did not follow a predictable dose response – a phenomenon we have previously observed. The ratio of blood to buffer needed to allow differentiation between RML-prion infected blood from healthy controls for both CD-1 and Tga20 mice was 1:10, whereas Sc237-prion infected hamster blood required a 1:1 ratio. Differences in chemiluminescense signals between experiments can be attributed to the heterogeneous nature of blood samples and to minor variations in materials and instrumentation that influence the magnitude of the arbitrary chemiluminescense values recorded on any given day.

### Detection in preclinical blood samples

**Figure 3:**
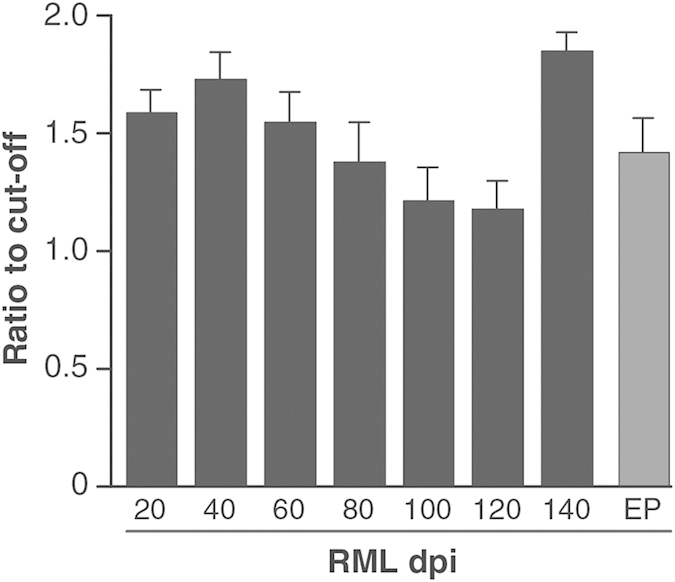
Detection of infection in preclinical blood samples. DDA signals from pooled blood samples taken at 20, 40, 60, 80, 100, 120, 140 dpi and clinical end-point (EP) from CD-1 mice infected with RML prions. Samples were analysed in triplicate in each of four independent assay runs. Data are shown as the mean chemiluminescent signal ratio relative to a cut-off (error bars indicating SEM) determined from the mean + 3× SD of control non-infected blood samples.

**Figure 4:**
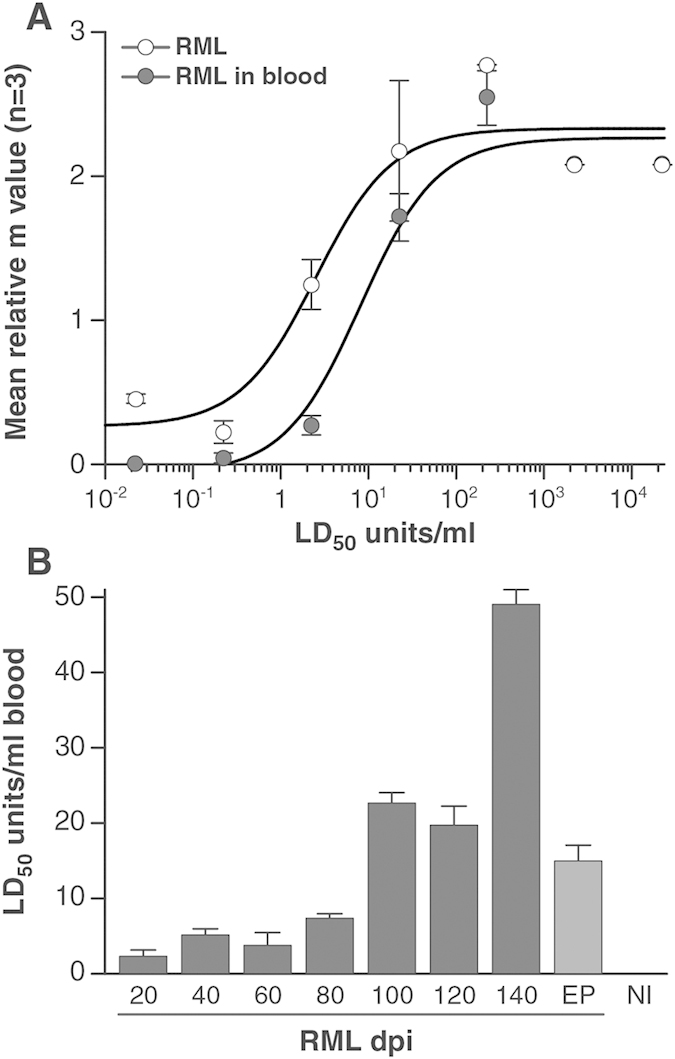
Detection of infectivity in blood prior to onset of clinical disease. Detection and quantification of infectivity in samples taken at 20, 40, 60, 80, 100, 120, 140 days post infection (dpi) and clinical end-point (EP) from CD-1 mice infected with RML prions was achieved using SSBA. Panel (**A**) shows the correlation between m value (n = 3, mean +/− SD) and titred RML-infected brain homogenate when spiked into either OFCS/10^−4^ FVB/N-*Prnp*^*0/0*^ (open circles) or control rodent blood (closed circles). Panel (**B**) shows the calculated LD_50_ units/ml in whole blood taken from mice culled at each of the 20 day intervals post inoculation with RML prions. The m value obtained in the SSBA for each of the time point groups (n = 12, mean +/− SD) was converted to LD_50_ units/ml using the correlation between m value and LD_50_ units/ml for titred RML-infected brain homogenate spiked into control rodent blood as shown in panel (**A**). The data were fitted to the equation m = (B + ((M*L)/(K + L))), where K = a saturation constant (mid-point of the m value amplitude), B = background m value and M = m value amplitude or range and L = prion titre.

**Figure 5:**
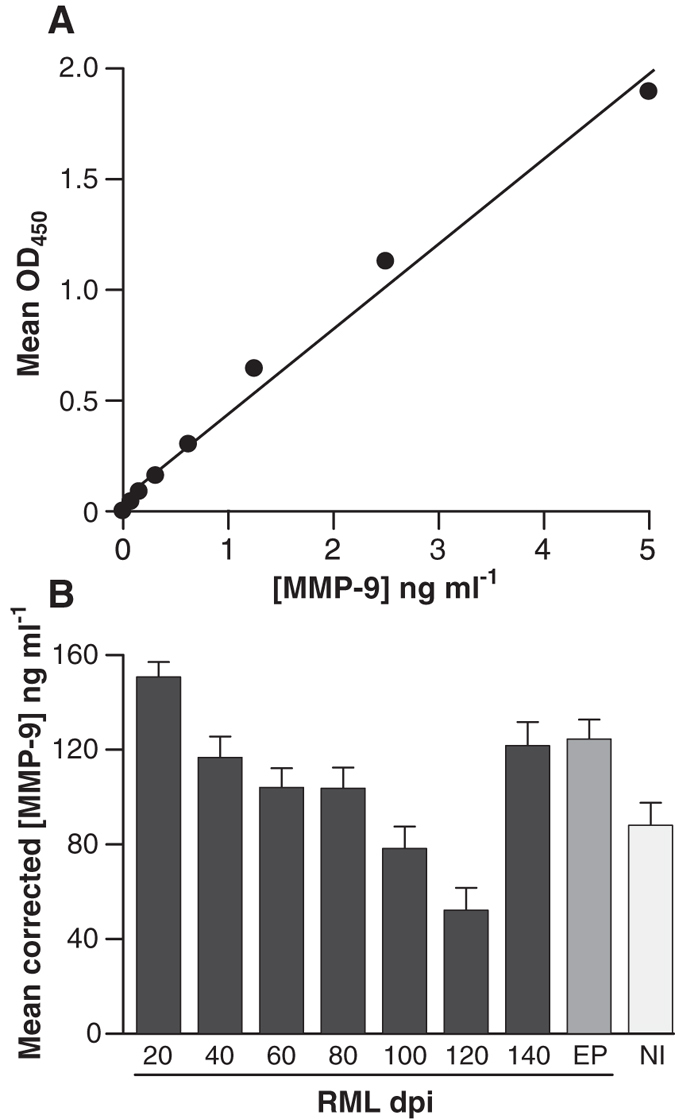
Assessment of blood-brain barrier integrity using MMP-9. Panel (**A**) shows the correlation between the mean OD_450_ nm readings and the concentration of a standard dilution series of MMP-9 (ng ml^−1^). This data was used as a standard curve for converting OD_450_ nm readings to the concentration of MMP-9 in blood. The data in panel (**B**) display the corrected mean (n = 3, mean +/− SD) concentration of MMP-9 in ng ml^−1^ in pooled whole blood from CD-1 mice inoculated with RML-infected brain homogenate at 20 day intervals post inoculation (dpi) until clinical end point of disease (EP). Pooled blood from non-infected control CD-1 mice is represented as NI.

To address the feasibility of detecting prion infection in sub-clinical states using the DDA, blood samples were taken from RML-prion infected mice at regular intervals through the incubation period until onset of clinical disease and compared to blood samples from non-infected control animals. Groups of ten CD-1 mice were inoculated intra-cerebrally with 1% w/v RML-prion infected end-point brain homogenate. Animals were then culled and blood samples taken at 20 day intervals post inoculation until the clinical end point of 157 (+/−8) days was reached. Blood samples from mice at each of the time points were pooled and analysed against healthy controls using the DDA (Fig. 3). Samples were analysed in triplicate in each of four independent assay runs. The mean chemiluminescence signals from each of the blood samples taken at 20 day intervals post inoculation had a ratio to cut-off (mean of control plus 3× standard deviations from the mean) greater than 1. Thus it is possible to differentiate preclinical infection in CD-1 mice infected with RML-prions throughout the incubation period and as early as 20 days post inoculation (dpi). The peak ratio to cut-off of 1.88 (+/−0.09) is observed in blood samples taken from mice 140 dpi, and is elevated relative to the signals obtained immediately prior at 120 days and at clinical end point (p < 0.05, unpaired t-test, two-tailed with Welch correction). A similar pattern of infectivity changes with time was observed with a distinct increase in infectious titre immediately prior to the onset of a clinical syndrome (Fig. 4B). The basis for this is not clear but may result from a sudden deterioration of the blood-brain barrier (BBB) releasing material into the bloodstream and periphery (Fig. 5), which is then diluted and cleared resulting in a return to previous levels.

### Detection of prion infectivity in blood prior to onset of clinical disease

To investigate the relationship between DDA signals and the quantitative detection of infectivity in these samples, blood taken from the time course incubation were also analysed using the SSBA. The SSBA utilizes the binding of prion infectivity to steel in combination with the highly prion-susceptible cell line PK1. It has a dynamic range of 6 logs and a sensitivity to 0.0029 (+/−0.002) TCIU_w_ 18 or less than 0.5 LD_50_ units/ml^22^. The level of infectivity measurable in the blood of these animals steadily increased with time, maximum titres being observed at 140 dpi, approximately 20 days prior to clinical end point of disease (Fig. 3), in close agreement with previously published results^23^,^24^. Remarkably, infectivity was clearly detectable at the earliest time point of 20 dpi, with a titre equivalent to 2.3 (+/−0.85) LD_50_ units/ml. The detectable titre of prion infectivity was found to rise with time to a maximum of 49 (+/−1.93) LD_50_ units/ml of blood of mice at 140 dpi. This finding of approximately 50 LD_50_ units/ml of prion infectivity in rodent blood is in close agreement with previous studies which estimated the prion titre in blood using rodent bioassay^25^,^26^,^27^,^28^. In common with the detection of abnormal PrP by DDA described above, this spike in infectivity occurred prior to the onset of clinical signs and levels returned to 14.9 (+/−2.1) LD_50_ units/ml in blood samples from mice at the clinical end-point of disease.

### Integrity of the blood-brain barrier over a time course following RML inoculation

The concentration of matrix metalloproteinase 9 (MMP-9) in serum is a potential indicator of BBB integrity^29^,^30^,^31^,^32^, with elevated concentrations indicating breakdown or increased permeability. Blood from the same groups of CD-1 mice analysed by DDA and SSBA were assayed for the concentration of MMP-9 using the commercial Mouse Total MMP-9 ELISA kit (Molecular Probes). Analysis of whole blood from RML-inoculated mice at 20 dpi demonstrated the presence of 152 (+/−6.7) ng/ml of MMP-9 compared to 94 (+/−12.5) ng/ml in uninoculated controls. The concentration of MMP-9 was observed to reduce with time until 120 days post inoculation (dpi) to 54 (+/−12.1) ng/ml, after which point the concentration increased to 125 (+/−13.7) ng/ml at 140 dpi and 122 (+/−10.1) ng/ml at the clinical point of disease (Fig. 4). The results are consistent with a breakdown of the BBB late in the incubation period, occurring at around 140 dpi or >85% of the incubation period.

### Correlation of DDA signal, levels of MMP-9 and infectivity in blood from RML prion-infected mice during incubation period to onset of clinical disease

In an attempt to gain insight into the progression of prion disease via rodent models and to understand how signals observed in our prototype human blood test (DDA), relate to prion infectivity found in blood, we correlated data obtained for each of the assays. It was found that there is a direct correlation between signals observed in the DDA assay and the level of MMP-9 in blood of RML inoculated CD-1 mice taken at 20 day intervals throughout the duration of disease until end point (P < 0.05; Pearson correlation, two-tailed test). However, neither the DDA signals nor the MMP-9 concentration in blood correlated with the measurable prion infectivity titre found in these blood samples as determined by the SSBA. However, detectable prion infectivity as determined by the SSBA analysis does show a significant positive correlation with time (Pearson correlation with p < 0.05), increasing throughout the incubation period.

## Discussion

The ability to accurately diagnose prion infection using biochemical methods is crucial to protect public health from iatrogenic transmissions, particularly arising from the transfusion of contaminated blood and blood products^10^,^11^,^12^,^33^. In the United Kingdom (UK) measures taken to prevent secondary infections of vCJD have included leucodepletion of donor blood, sourcing of plasma from non-UK sources, exclusion of transfusion recipients from donation, use of recombinant clotting factors for patients with bleeding disorders and ceasing the use of UK plasma for fractionation and export^34^. We have previously reported the development^19^ and validation^20^,^35^ of a blood test for vCJD based upon the detection of abnormal disease-specific isoforms of PrP, which is now in clinical use for the diagnosis of prion disease. The wider application of this test, or indeed other emerging assays^36^,^37^, for screening asymptomatic individuals for potential infection is confounded by an inability to confirm that an assay detection limit is sufficient for the detection of preclinical infections. This problem arises as there are currently no samples available from individuals confirmed as sub-clinical carriers of vCJD with which to assess either the prevalence of prionaemia in BSE prion-exposed populations or the sensitivity of any potential blood tests. A definitive answer will require prolonged longitudinal study of individuals testing positive to determine what proportion of patients with vCJD prionaemia go on to develop clinical vCJD and how many are chronic carriers^38^.

It has been suggested that carriers would have lower concentrations of abnormal PrP compared to individuals with clinical CJD^13^, and this seems likely. Despite potentially lower levels, animal models indicate clear preclinical blood involvement^23^,^24^,^39^,^40^ and very efficient transmission of prion infection has been demonstrated from blood taken from sheep in early preclinical stages of scrapie^9^. The use of the RML-prion strain allows rapid and precise estimates of low prion titre using cell-based assays^41^,^42^. Previous attempts to detect infectivity in low titre sources have required either the use of large numbers of recipient animals or the transfusion or large volumes of infected analyte in large animals. By using the SSBA^18^ we have not only verified but accurately quantified the presence of infectivity in blood even at the earliest stages of preclinical prion disease. A comparison of RML-infected brain diluted into either FVB/N-*Prnp*^0/0^ brain homogenate or blood showed that although the presence of whole blood impacted on the overall detection limit of the assay, it remained capable of detecting RML prions at concentrations of less than 1 LD_50_ units/ml. Infectivity was found to rise by approximately 10–20-fold throughout the incubation period (Fig. 4) as might be anticipated from historical estimates derived from conventional large scale rodent bioassays^26^, albeit with significant fluctuations approaching the clinical end-point of disease. The significant increase in infectious titre in the last few days of the incubation period are unexpected with the change in titre identifiable only as a result of the high precision of SSBA and related cell-culture assays which are providing unique insights into the replication of prion isoforms during pathogenesis^43^,^44^. Levels of MMP-9 in serum provide an indication of BBB integrity and although complicated by age-related decline^45^, sudden elevation towards the clinical end point for prion disease (Fig. 5) may indicate a sudden perturbation leading to exchange of prion material between the CNS and circulating blood and fluctuations in the concentration of prion disease-related PrP isoforms in blood.

Surprisingly, analysis of the same samples using our DDA assay revealed relatively consistent positive signals throughout the RML incubation period (Fig. 3). Positive detection was achieved from the earliest time point sampled and could not be ascribed to the detection of residual inoculum as similar signals were not observed in the blood of FVB/N-*Prnp*^0/0^ mice unable to replicate prions (data not shown), and the half-life of prion-infected inocula has previously been shown to be short: 36 hours in rodent brain tissue^46^ and less in cell-culture^47^. The lack of correlation between DDA reactivity and infectivity seen by SSBA indicates DDA is capable of detecting a wider ensemble of abnormal PrP conformers associated with prion infection^44^,^48^ of which infectivity may only constitute a minority component. Such observations are not without precedent and there is increasing evidence to suggest that abnormal PrP conformers may be as much as 10^6^-fold more abundant than assayable infectivity^49^. The ability to detect the plethora of abnormal PrP increases the analytical sensitivity of assays and in the case of DDA an analytical sensitivity equivalent to a 10^9^-fold dilution of prion-infected brain is sufficient for clinical sensitivity at the earliest stages of preclinical prion disease.

## Materials and Methods

### Research governance

Work with prion-infected samples was conducted in microbiological containment level 3 facilities with strict adherence to safety protocols. Work with animals was performed in accordance with licences approved and granted by the UK Home Office (Project Licences 70/6454 and 70/7274) and conformed to University College London institutional and ARRIVE guidelines.

### Animal experiments

RML or Sc237 prion inocula were prepared as a 10% (w/v) brain homogenate in Dulbecco’s phosphate buffered saline lacking Ca^2+^ or Mg^2+^ ions (D-PBS)^50^. Inocula were diluted to 1% (w/v) with D-PBS and 30 μl inoculated intracerebrally into experimental animals. Normal CD-1, Tga20 mouse or Syrian hamster brain homogenate (1% w/v) was inoculated similarly into control animals. Animals used were outbred wild-type CD-1 and Tga20 transgenic mice^21^ (backcrossed to FVB/N mice; eightfold over expression of wild-type PrP^C^), and wild-type Syrian hamsters. Groups of animals (female 6–9 weeks old) were inoculated and monitored as described previously^43^. For the analysis of blood from animals at the clinical end point of prion disease, groups of 20 were euthanized and whole blood obtained by cardiac puncture for pooling in EDTA tubes. Control materials were obtained in an identical manner from age-matched uninoculated animals. To obtain samples from defined time points during the incubation period for disease, groups of 30 RML prion inoculated CD-1 mice were euthanized at intervals of 20 days and blood pooled into EDTA tubes. The final concentration of EDTA in all samples was in the range 6–12 mM. All samples were stored frozen at −70 °C.

### Direct Detection Assay (DDA) for prion infection

The detection of abnormal, disease-specific isoforms of the prion protein was performed with modifications to the method previously described^19^. Briefly, experiments involving the exogenous spiking of RML-prion infected brain homogenate into whole blood obtained from wild-type CD-1 mice utilised a dilution ratio of 1:10 into assay capture buffer (200 mM Tris pH8.4, +4% w/v BSA + 4% w/v CHAPS + 2× concentration of Complete protease inhibitors [Roche, Mannheim, Germany]). Assay read out was in arbitrary chemiluminescence units. For comparison of signal levels across the incubation period for prion disease this was normalised to a ratio relative to cut-off (RRC) with the cut-off threshold defined as the mean signal from non-infected controls + 3× standard deviations from that mean. Hence values greater than one indicate the presence of infection.

Exogenous spiked samples were prepared by serial dilution in whole blood from an initial concentration of 1% w/v brain homogenate.

Exploration of optimal dilutions for varying strains of prions used endogenous blood samples obtained from wild-type CD-1, transgenic Tga20 mice^21^ or Syrian hamsters taken at the clinical end-point of prion disease following inoculation with RML or Sc237 prions respectively. Dilutions of 1:1, 1:10 and 1:100 in capture buffer were tested and compared to control samples from equivalent uninfected animals.

### Detection of infectivity in rodent blood

Infectious RML prions in blood were quantitatively detected using the Standard Steel Binding Assay (SSBA)^18^. Briefly, monofilament Steelex® wires were exposed to prion infected samples for 3 hours before being rinsed several times in PBS, and dried. Up to 20 wires were placed into each well of a 6-well cluster plate (Corning) and covered with a suspension of 3 × 10^5^ N2aPK1^41^ cells in 5 ml OFCS (Opti-MEM [Invitrogen] + 10%v/v FCS + 1% w/v penicillin/streptomycin). After culture for 3 days at 37 °C and 5% CO_2_, the wires with adherent cells were transferred into fresh wells containing 1 ml OFCS. The adherent cells were dislodged from the wires by vigorous pipetting, collected in a separate tube, and counted. Cells were seeded into wells of 96-well plates (Corning) and after 3 days split at a ratio of 1:3 before being split 1:8 into fresh cell culture media and grown back to confluence. Two further splits were conducted before transferring a sample of the cells to ELISPOT plates for analysis of the number of cells containing PK-resistant PrP and analysis by SCEPA^41^,^42^.

Endogenous whole blood samples taken from CD-1 mice at defined time points in the incubation period for disease were analysed at a 1:10 dilution of blood to buffer (80 μl blood was diluted in 720 μl capture buffer). To create reference curves wires were exposed to two logarithmic dilution series (10^−4^ to 10^−10^) of RML-infected brain homogenate; one prepared in 10^−4^ FVB/N-*Prnp*^*0/0*^ brain homogenate in OFCS and one in whole blood from uninfected CD-1 mice.

### Tissue Culture Infectivity Units and correlation with LD_50_ Units

SCEPA allows the calculation of an ‘m’ value, the average number of infectious units (either in the form of infected cells or prions) delivered into a well, using the Poisson equation (*P*_(0)_ = *e*^−*m*^, where P_(0)_ is the probability of a well remaining uninfected) i.e., non-infected wells/total number of wells. Thus, if a certain amount of a sample were to give 9 uninfected wells from a total of 24 receiving sample, then it would contain on average m = ln (24/9) = 0.98 or about one infectious unit which, in the case of the wire-mediated assay under standard conditions, we have previously defined as a TCIU wire unit (TCIU_w_)^18^. We can establish a relationship between LD_50_ units/ml (as established by the mouse bioassay) of the solution to which the wires were exposed, and m value by exposing wires to a serial dilution of RML-infected brain homogenate with a known rodent titre from bioassay and determining m values resulting from the SSBA.

### MMP-9 Assay

The levels of the matrix metalloproteinase-9 (MMP-9) in the blood of CD-1 mice were assayed using a commercially available reagent (Mouse total MMP-9 ELISA kit (Molecular Probes)).

## Additional Information

**How to cite this article**: Sawyer, E. B. *et al.* Preclinical detection of infectivity and disease-specific PrP in blood throughout the incubation period of prion disease. *Sci. Rep.* **5**, 17742; doi: 10.1038/srep17742 (2015).
